# An effort to enhance the clinical translatability of caprate-based tablet formulations in gastric peptide delivery

**DOI:** 10.1007/s13346-025-01978-7

**Published:** 2025-09-23

**Authors:** Pierre-Louis Bardonnet, Zhigao Niu, Jenni Pessi, Maria Kazakou, Konstantinos Raptis, Reece McCabe, Anders Toftlev, René Rebollo, Zhuoran Wang, Li Fan, Nicolai Rytter Mortensen, Lars Bardtrum, Vincent Andersson, Per-Olof Wahlund, Mathias Norrman, Andrew James Benie, Jian Xiong Wu, Max Sauter, Damiano La Zara, Philip Christophersen, Philip Jonas Sassene

**Affiliations:** 1 Novo Nordisk A/S, Global Research Technologies, Måløv, 2760 Denmark; 2 Novo Nordisk Research Centre China, Beijing, 102206 China; 3 Novo Nordisk A/S, Clinical Drug Development, Søborg, 2860 Denmark

**Keywords:** Oral peptide delivery, Permeation enhancer, Sodium caprate, Preclinical, Clinical

## Abstract

Sodium caprate (C10) is the most investigated permeation enhancer to promote oral peptide absorption. However, the clinical translation of C10-based formulations is possibly affected by low gastric pH. Here, we developed a C10-based immediate-release tablet containing meglumine as a pH modifier to mitigate stomach acidity and evaluated it both in dogs and clinically. To mitigate the difference in gastric pH between species, the C10-based formulations were evaluated in acid pre-treated dogs. The exposure was compared to results with sodium salcaprozate (SNAC)-based tablets previously tested in clinical trials. The benefit of meglumine in improving gastric peptide absorption in dogs was demonstrated for several peptide modalities. Ultimately, an oral PCSK9 inhibitor was chosen for test clinical trials. The lead formulation containing 40 mg of PCSK9 inhibitor, 200 mg of C10, 60 mg of meglumine and 60 mg of sorbitol showed a 57% increase in exposure compared to the benchmark SNAC formulation in animal studies 0.5 h post dosing. However, this benefit was not observed in humans to the same extent, where the C10-based formulations provided similar bioavailability to the SNAC-based formulation. Other factors than pH which are likely to influence the relative performance of C10- and SNAC-based formulations are also discussed in this article.

**Graphical abstract:**

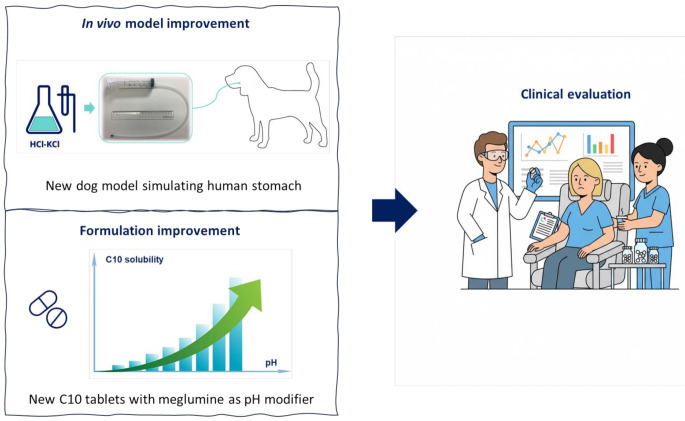

**Supplementary Information:**

The online version contains supplementary material available at 10.1007/s13346-025-01978-7.

## Introduction

Peptide and protein drugs play an important role in several therapeutic areas in modern medicine, such as cardiometabolic diseases, oncology, immunology, and cardiovascular diseases, covering a growing number of patients worldwide. The high potency and specificity of these macromolecules offer great clinical advantages over traditional small molecules, yet their complex structures and labile nature in the gastrointestinal tract complicate their oral administration [1]. Although injectables remain the most common administration form for peptides and proteins, oral tablets are preferred by patients because of their convenience of administration [2]. Moreover, an oral modality may provide additional benefits in terms of safety, cost-effectiveness, and shelf-stability.

Extensive academic and industrial research has been carried out towards the development of oral peptide and protein formulations containing fatty acids or fatty acid-derived permeation enhancers (PE) such as salcaprozate sodium (SNAC), sodium caprylate (C8) and sodium caprate (C10). These efforts have led to the successful FDA registrations of SNAC-based oral semaglutide and C8-based oral octreotide [3–6]. The fatty acid PEs temporarily opens the intercellular tight junctions in the gastrointestinal epithelium and increase membrane fluidity [5, 7], thereby increasing the permeability of these drugs. Formulations containing C10 has shown potential in preclinical studies, but its translation into clinical use is currently limited due to challenges such as variable absorption, supply and processability issues. While C10 has mainly been studied as PE for intestinal delivery [8–11], recently Tran et al. reported the use of C10 for gastric delivery of a GIP/GLP-1 peptide [12]. Specifically, C10 was found as effective as SNAC in enhancing the gastric absorption of the peptide orally administered as an uncoated tablet in monkeys. For immediate-release oral peptide formulations, the stomach is the main site for tablet disintegration, dissolution and absorption, particularly in the case of molecules sensitive to intestinal proteases which would degrade once in the duodenum. Medium chain fatty acids can also be absorbed in the stomach [13, 14] as they are not incorporated into chylomicrons and can be absorbed by passive diffusion to the portal vein [15, 16].

**Fig. 1:**
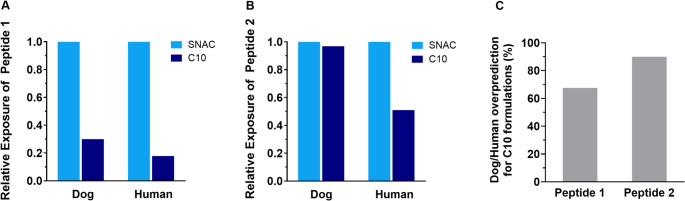
Relative exposure of C10 tablet formulations versus SNAC tablets for acylated peptides after oral administration to dogs and humans, respectively. (**A**) and (**B**) indicate the normalized exposure of two different in-house made peptides in C10-based tablets against in SNAC tablets. (**C**) indicates the overprediction (%) of exposure in dogs versus humans for the C10-based formulations

Inadequate animal models for validating new formulation concepts represent a barrier for successful translation of C10-based formulations. Most in vivo readouts reported in literature are from rodents, where a poor correlation with clinical data is unsurprising considering the physiological differences between species. Moreover, even when larger animals such as dogs and pigs are employed due to the resemblance of their gastrointestinal (GI) tracts to humans [6], translational issues may arise in the case of C10-based formulations: for instance, our unpublished in-house data show that the exposure of two internal acylated peptides in C10-based formulations dosed in naive beagle dogs systematically overpredicts their clinical bioavailability (Fig. 1). A potential reason for the lack of translation may be the discrepancy in gastric pH between dogs and humans. In the fasted state, human gastric pH is reported to be around 2 for healthy volunteers [17–19], while beagle dog stomach commonly has higher and more variable pH [20, 21], with reported values ranging from 3 to 7 [22, 23]. The pH variability in dog stomach may be explained by a more significantly fluctuating gastric secretion rate in the fasted state, likely due to higher gastric responsiveness upon stimulation [24, 25]. Different gastric environments between species may affect tablet performance, ultimately altering bioavailability.

In this work we developed a novel C10-based tablet formulation containing a pH modifier and evaluated it preclinically in beagle dogs pre-treated with acid to reflect the human gastric pH. Different active pharmaceutical ingredients (APIs) were used for the preclinical exploration, and the one selected formulation was ultimately evaluated in a clinical trial.

## Results and discussions

### Acidic dog model reflecting the human stomach

**Fig. 2:**
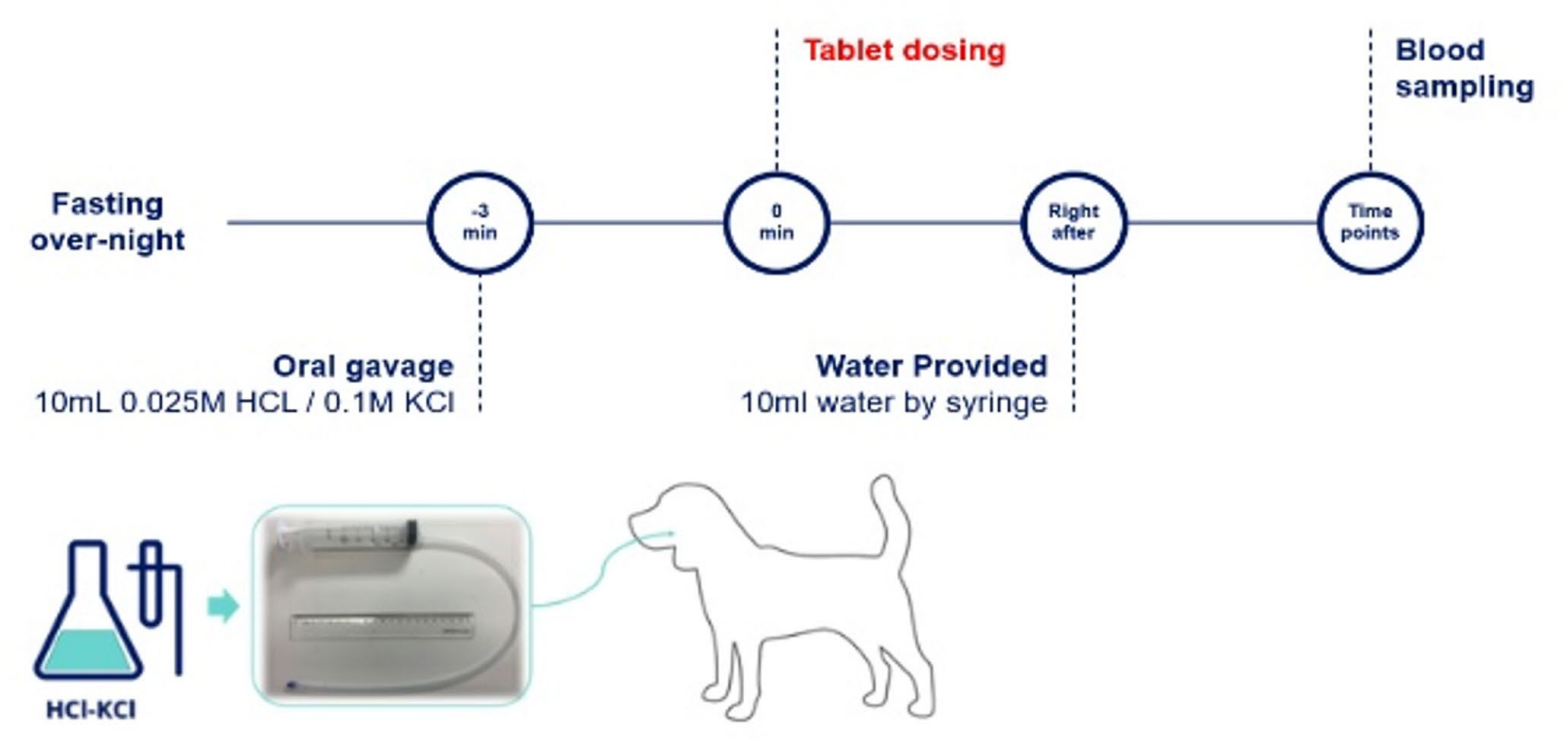
An illustration of the in vivo dosing sequence in gastric pH adjusted beagle dogs. After animal being fasted overnight, HCl-KCl solution is administrated by oral gavage 3 min prior to tablet dosing. 10 mL of water is administrated right after the tablet dosing

The gastric pH in dogs was artificially reduced to match that of humans. Acidification of the dog stomach can typically be achieved by pentagastrin injections or oral dosing of acidic solutions. While pentagastrin administered by intravenous or sub-cutaneous injections can increase acidic secretion in the stomach, it can also induce secretion of pepsin [26, 27] as well as impact gastric motility and gastric emptying [22], thereby introducing additional biases. In contrast, by delivering an acidic solution via oral gavage, only the gastric pH is impacted. Analysis of the gastric fluid content in in-house beagle dogs showed an average amount of 0.3 ± 0.1 g/kg (*N* = 4, average body weight of 11 kg), comparable to the 0.47 ± 0.52 g/kg found by Mori et al. [21]. As the body weight in the dog colonies spans from 10 to 16 kg, a fixed amount of 10 mL of HCl solution was selected to standardize the procedure, leading to a volume of fluid per kg of body weight varying from 0.9 to 1.5 mL/kg, which is in accordance with the gastric fluid volume of up to 1.5 mL/kg found by Perlas et al. in humans [28]. The concentration of HCl was set to 0.025 M (pH 1.6) to reach a final pH between 1.7 and 1.8, once mixed with the fluid already present in the dog stomach (Table S1). Osmolarity was adjusted by adding 0.1 M KCl in the oral gavage solution and administered 3 min before oral dosing of the tablet to ensure an acidified dog stomach. The dosing sequence is illustrated in Fig. 2.

An in vivo evaluation was conducted with SNAC-based and C10-based tablet formulations to validate the hypothesis that the dog model with humanized gastric pH level would increase the translatability of C10 formulations for gastric peptide delivery. A glucagon-like peptide 1 (GLP-1) analogue (Figure S1) was used as model peptide for test. To enhance animal welfare, the study prioritized the initial 30 min of the pharmacokinetic (PK) curve, minimizing the frequency of blood sampling. This timeframe is also particularly pertinent for gastric peptide delivery in clinic, since these formulations exhibit high sensitivity to food intake and essentially no absorption in the postprandial state [29, 30]. Indeed, the tablet should be taken on an empty stomach with water, followed by a waiting period of at least 30 min before eating, drinking, or taking any other oral medicinal products. Consequently, this PK parameter has been selected as a surrogate of bioavailability in our study.

**Fig. 3:**
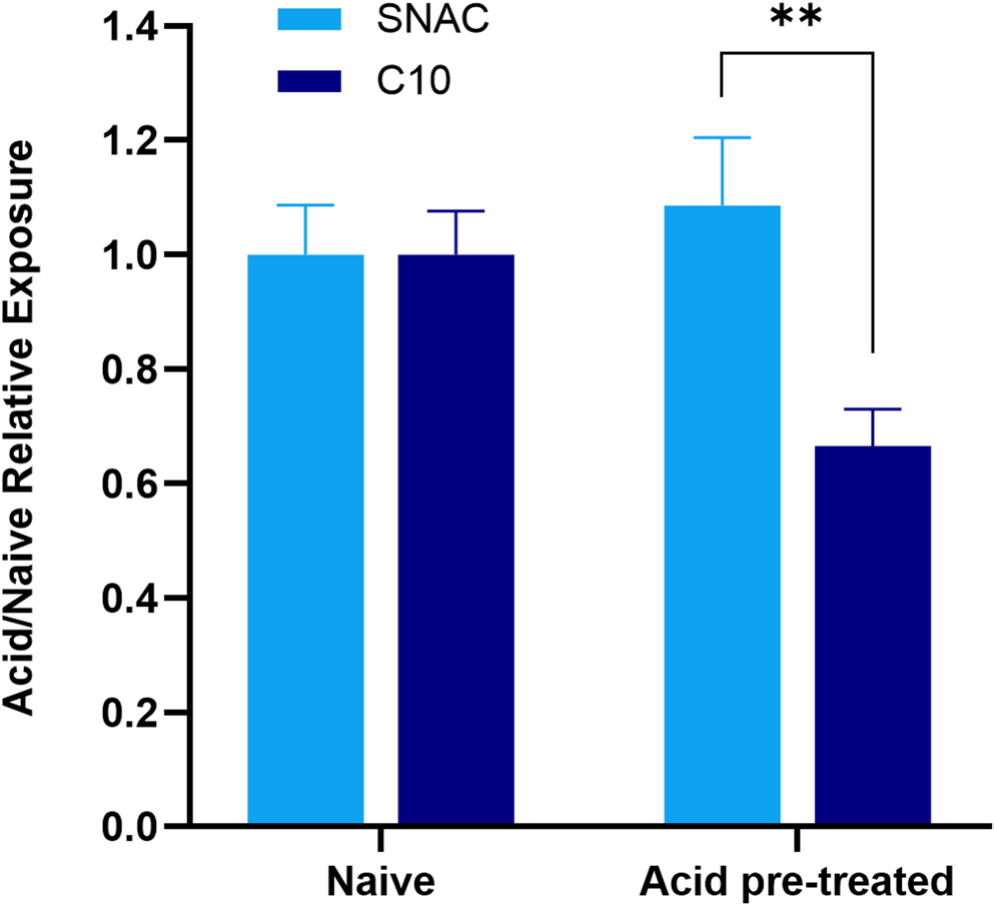
Normalised dose-corrected plasma exposure (Cp/D) of the GLP-1 analogue in naive dogs and in acid pre-treated dogs, at 0.5 h after oral dosing of the SNAC tablets and the C10-based tablets. The Cp/D for naive dogs is deemed as 1. Data expressed as mean ± SEM, *N* = 16 (except for the SNAC-based formulation dosed in naive dog where *N* = 40). In each animal model, the mean value of C10 formulation was compared with that of SNAC formulation by a two-tailed unpaired t-test. ** *p* < 0.01

As shown in Fig. 3, the dose-corrected plasma exposure (Cp/D) at 0.5 h for the SNAC tablets was comparable in the naive dogs and acid pre-treated dogs (*N* = 16, otherwise stated). In contrast, the Cp/D of C10-based tablet formulation showed a 33% decrease in the acid pre-treated dogs compared to in the naive dogs, at 0.5 h post-dosing of the same GLP-1 peptide. This drop is consistent with the bioavailability reduction previously observed for C10-based tablets in the clinical trials (Fig. 1). Accordingly, the agreement between data from the preclinical acidic dog model and the clinical data supports the hypothesis of differing gastric pH values being accountable for the lack of translation between dogs and man. The acid-pretreated dog model was selected as in vivo model for further optimization of C10-based formulations.

### Incorporation of a pH modifier for improved tablet performance

**Fig. 4:**
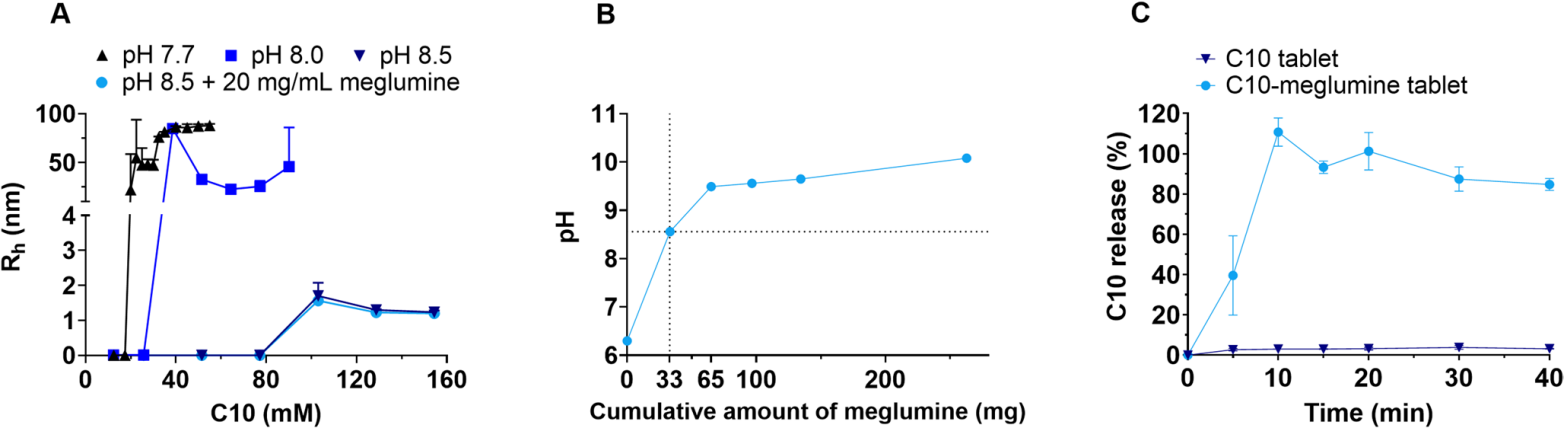
Physicochemical characteristics and pH of C10 and meglumine mixtures. (**A**) Buffering capacity of meglumine in 20 mL of acidic medium (0.0125 M HCl containing 5 mg/mL of C10); (**B**) Hydrodynamic radius of C10 at different pH values as a function of C10 concentration; and (**C**) C10 release profile from tablets with and without meglumine in 20 mL media (initial pH 1.9) mimicking gastric conditions. Data expressed as mean ± SD, *N* = 3

Given the negative impact of low gastric pH on both in vitro and in vivo performance of C10-based tablets, the use of a pH modifier was investigated to mitigate the acidity of the human stomach. Since C10 is an ionizable compound known to form different colloidal structures dependant on pH, selection of an appropriate pH modifier is paramount. The resulting pH should enable fast disintegration and dissolution of the formulation, whilst securing that the colloidal species formed facilitate the permeation enhancing effect needed for absorption of the macromolecule. Monomeric C10 is presumed to be the primary form needed to partition across the mucus layer to the epithelium and as well as exerts its effect on epithelial membrane and intracellular tight junctions. Above pH 8.5, C10 predominately forms micelles (Fig. 4A) and shows a critical micelle concentration (CMC) increasing with pH from 81 mM at pH 9.1 to 102 mM at pH 11.8 [31]. Therefore, at higher pH there should be more monomeric C10 present. Moreover, the equilibrium between C10 micelles and monomers in solution is very rapid, with an exchange typically occurring in a time range of nano- to microseconds [31]. At pH values lower than approximately 8.5, vesicles start forming with a critical aggregate concentration (CAC) of around 15 mM (Fig. 4A) and at even lower pH values, oily droplets are also formed. The presence of vesicles or droplets also results in an inherently slower exchange of C10 with the ‘free’ monomeric species, consequently when C10 is removed from the system (e.g. by partitioning to the membrane or binding to tight junctions) it is replenished at a significantly slower rate. These characteristics are in line with the lower performance of C10 formulations in the more acidic human stomach compared to dogs. For this reason, we hypothesize that a pH modifier securing pH elevation to approximately 8.5 may be beneficial in C10 formulations.

On top of elevating the pH, the pH-modifying excipient needs to ensure manufacturability of tablets while being compatible with the API in the formulation. An array of alkaline agents capable of increasing the pH were screened (Figure S2) by addition of the pH modifier in 20 mL of acidic media (0.1 M HCl) with 0.1 M KCl, and 10 mg/mL of C10 at 37 °C, which is close to the physiological condition of experimental animals when C10 tablet formulation is dosed. The volume and concentration of HCl and KCl were selected to replicate the gastric in vivo conditions, whereas C10 was added to mimic the intrinsic buffer capacity of the PE present in the tablet. Many of the pH modifiers tested proved unsuitable for the purpose, as they were unable to increase the pH to a desired level when a practical amount was employed in a tablet formulation. Strong bases such as NaOH and KOH were also unsuitable due to their hygroscopicity and incompatibility in producing tablets meeting technical requirements. Alkaline salts with divalent or trivalent cations were discarded due to the potential formation of insoluble soaps with C10. Sodium carbonate was chosen as the first candidate; however, it led to adverse events (i.e. vomiting) in dogs, possibly associated with potential gas formation upon reaction between sodium carbonate and gastric acids. The drastic pH increase induced by sodium carbonate (Figure S2) may also induce potential mucosa damage in the stomach.

Meglumine was selected due to its capability to both raise the pH to the desired level and produce tablets with the required quality. In fact, meglumine has a pKa of 9.6 and is therefore appropriate for buffering the pH around 9.6 ensuring that the pH can be raised to above 8.5 and kept in a suitable range even for varying gastric volumes and acidity. In addition, it is an approved pharmaceutical excipient marketed in several oral dosage forms such as telmisartan, repaglinide and rosuvastatine tablets. To identify the most suitable meglumine amount for C10-based tablets, a systematic screening was conducted in 20 mL acidic medium (KCl 0.1 M/ HCl 0.025 M, 10 mL + 10 mL of MQ water) reflecting the composition of dog gastric fluid after dosing of water. The medium contained either 50, 100, 200, or 300 mg of C10 to account for different enhancer amounts, and the evolution of pH against added meglumine amount was monitored (Table 1). Since a final pH above 8.5 around the tablet in the stomach is desirable for C10 dissolution and to achieve the single-phased micellar system with high monomeric C10 availability, the corresponding meglumine amount needed to reach pH 8.5 was set as the lower limit for formulation development. For tablets with 100 mg C10, at least 33 mg meglumine is required (Fig. 4B). Due to the buffering of meglumine around its pKa of 9.6, higher amounts do not result in pH values significantly deviating from 9.6 and can therefore be adopted for formulation development.

Incorporating meglumine in the C10 tablets substantially improved the dissolution performance of C10, which was assessed in vitro in a small volume dissolution analysis mimicking physiological conditions. As shown in Fig. 4C, the formulation containing 50 mg of meglumine in tablets with 50 mg C10 resulted in a complete C10 release, compared to pure C10 tablets where almost no C10 was dissolved, clearly demonstrating the benefits of pH modulation by meglumine in immediate release tablets. Furthermore, meglumine did not show any impact on the CMC of C10 demonstrating its compatibility with the intended purpose of the formulation (Fig. 4B). Simultaneously, buffering the pH around the tablet would inhibit the conversion of pepsinogen to pepsin, and thereby protecting the released peptide from enzymatic degradation in the microenvironment in the stomach.

**Table 1:** Amount of meglumine required to adjust to pH 8.5 and 9.5 at different C10 concentrations

C10 / Fasted gastric fluids	50 mg /20 mL	100 mg / 20 mL	200 mg / 20 mL	300 mg / 20 mL
Amount of meglumine (mg) to pH 8.5	15	33	60	90
Amount of meglumine (mg) to pH 9.5	25	60	100	125

### Preclinical evaluation of lead formulations in acidic dog model

**Fig. 5:**
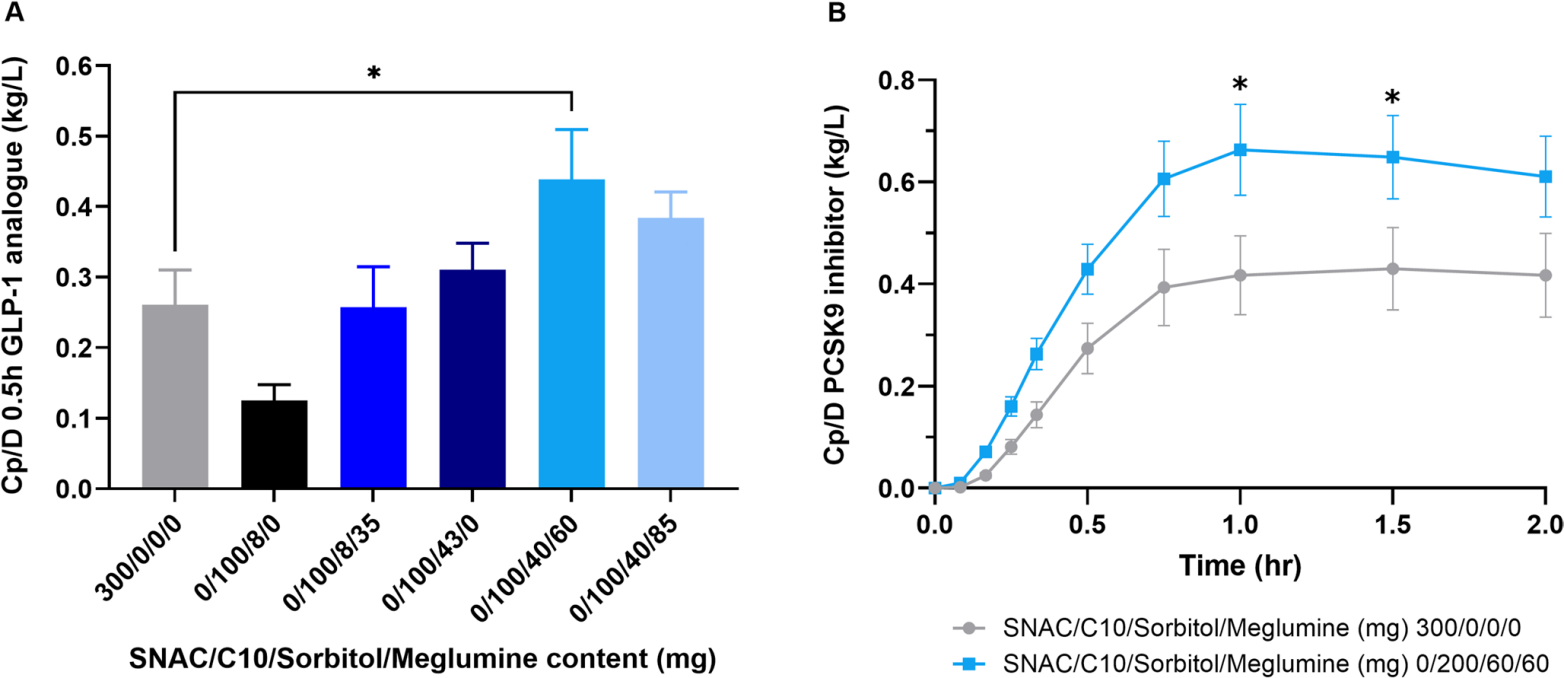
Dose-corrected plasma exposure (Cp/D) of GLP-1 analogue and PCSK9 inhibitor peptide after oral dosing of different tablet formulations to acid pre-treated beagle dogs. (**A**) Cp/D at 0.5 h post dosing of C10/sorbitol/meglumine formulations against different controls. From left to right: reference tablets made with SNAC only, control tablets with C10 only, control tablets with C10/meglumine, control tablets with C10/sorbitol, lead formulation with C10/sorbitol/meglumine, and C10/sorbitol/meglumine formulation with a higher meglumine content. A minimum amount of 8 mg of sorbitol per tablet was found to be necessary for manufacturability. (**B**) Cp/D of PCSK9 inhibitor over time after oral dosing of SNAC tablets and C10/sorbitol/meglumine based formulation. Data expressed as mean ± SEM, *N* = 16. The mean value of each column in (**A**) was randomly compared with that of the first column by one-way ANOVA. The mean value of each time point in (**B**) was randomly compared with that of the other curve by two-way ANOVA with Bonferroni’s correction. * *p* < 0.05

To ensure tablet manufacturability, the addition of sorbitol to the formulation containing C10, meglumine and lubricant was evaluated. The same GLP-1 analogue was used as model API at a dose of 5 mg/tablet. Specifically, two levels of sorbitol were selected as a hydrophilic filler for further investigation, with or without the addition of meglumine (Table S2). The C10/sorbitol/meglumine 100/8/35 showed a dose corrected plasma concentration after 30 min of 0.26 ± 0.06 kg/L, 2 times higher than the C10/sorbitol 100/8 (0.13 ± 0.02 kg/L) (Fig. 5A). However, when the sorbitol amount was increased from 8 to 43 mg, another control group matching the total sorbitol and meglumine amount in one tablet, the C10/sorbitol control formulation presented Cp/D of 0.31 ± 0.04 kg/L at 0.5 h, comparable to that of C10/sorbitol/meglumine 100/8/35 (Fig. 5A). This is likely due to the benefit of sorbitol with respect to tablet disintegration and dissolution. Two amounts of meglumine, 60 and 85 mg, were then tested in combination with C10 and 40 mg sorbitol, exhibiting a Cp/D of 0.44 ± 0.07 kg/L and 0.38 ± 0.04 kg/L at 0.5 h, respectively (Fig. 5A). In other words, tablets with C10/sorbitol/meglumine ratio 100/40/60 and 100/40/85 provided 42% and 23% improvement in GLP-1 exposure compared to the control tablets with C10 and sorbitol only. Moreover, when compared to the formulation comprising 300 mg SNAC, the C10/sorbitol/meglumine formulations displayed a 1.69- and 1.46-fold increase in GLP-1 peptide absorption. Based on the superior exposure, 60 mg of meglumine was thus selected as the lead formulation for clinical evaluation.

Ultimately, we adapted the formulations to a PCSK9 inhibitor, an LDL cholesterol lowering peptide, for clinical evaluation against a SNAC-based formulation. It is an acylated linear peptide with a molecular weight close to 6 kDa (Figure S1). To accommodate the clinically required higher dose of PCSK9 inhibitor, i.e. 40 mg, the amount of C10 was increased up to 200 mg in each tablet. Higher amounts of C10 (e.g. 300 mg) significantly delayed tablet disintegration from less than 30 min to over 70 min. Two different levels of meglumine were tested, 60 mg as the minimal amount required to reach pH 8.5, and 100 mg to ensure the buffering around pH 9 in the stomach. The sorbitol amount was also increased to 60 mg to ensure manufacturability and tablet performance. Prior to the clinical evaluation, these C10-based tablets were tested in acid pre-treated dogs benchmarking the SNAC formulation, where the lead formulation containing 200 mg C10, 60 mg of meglumine and 60 mg of sorbitol showed a 57% increase in exposure at 0.5 h post dosing (Fig. 5B). These results corroborated that having meglumine as a pH modifier in C10 tablets is not only advantageous for the gastric delivery of GLP-1 analogue, but also a strategy for delivering other therapeutic peptides. In addition, it is plausible that pH modifiers such as meglumine could also improve the performance of other permeation enhancers than C10, which have pH related solubility issues.

### Clinical evaluation of the C10/Meglumine formulation

**Fig. 6:**
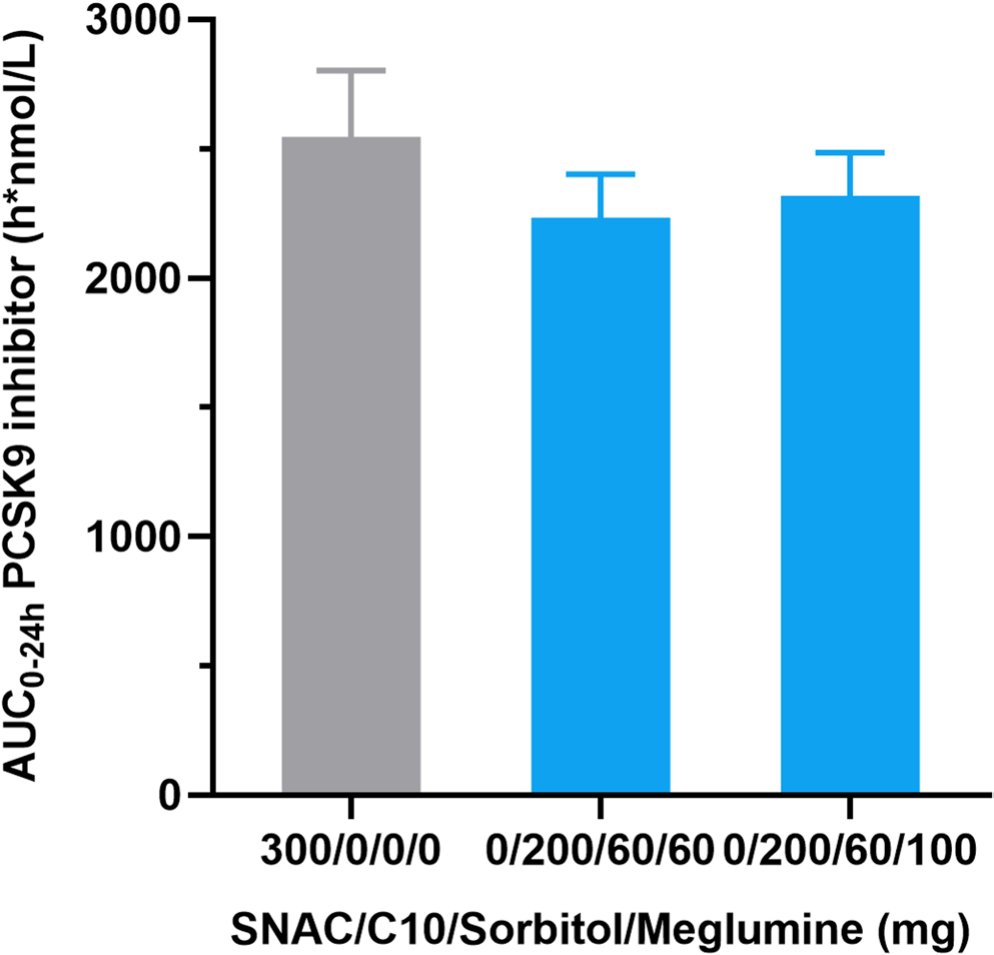
AUC_0 − 24 h_ of PCSK9 inhibitor in SNAC-based tablets and C10/sorbitol/meglumine-based tablet formulations. Data expressed as geometric mean ± SEM. *N* = 49 for tablets with 300 mg SNAC and tablets with C10/sorbitol/meglumine 200/60/60 mg, and *N* = 48 for tablets with C10/sorbitol/meglumine 200/60/100 mg

Three formulations, namely tablets with 300 mg of SNAC, C10/sorbitol/meglumine 200/60/60 or 200/60/100 mg, were evaluated in a clinical trial with PCSK9 inhibitor (40 mg). On par exposure with the SNAC formulation was obtained for C10 tablets with both low and high meglumine levels (60 mg and 100 mg respectively, Fig. 6). In clinic, this is the first time that we have observed on par exposure of a therapeutic peptide tested in SNAC tablets versus in C10-based formulations. Previous clinical trials (Fig. 1) unanimously showed considerably lower peptide exposure when C10-based formulations were employed. These results suggest that, despite the differences in the primary mechanisms of action between SNAC and C10, i.e. transcellular versus paracellular transport, both may equally contribute to enhanced oral peptide absorption. C10, which has been recognized as an intestinal permeation enhancer, could also play a role in the gastric lumen when formulated appropriately, as evidenced by the early onset of action observed in our studies.

Nonetheless, despite the enhanced performance of C10-based tablets over SNAC tablets observed during preclinical development, this advantage did not translate in humans. The dog model, which was designed to mimic human gastric pH levels, still proved inadequate in predicting the clinical performance of C10-based formulations, resulting in a significant overprediction for the in vivo performance of the C10-based tablets.

The persisting translational discrepancy in exposure between acid pre-treated dogs and humans for the C10 formulation may be explained by physiological differences between the two species and/or by inappropriate meglumine amount. Even if the local acidic pH was mitigated by meglumine, the paracellular absorption in human may be significantly limited by the tight junction physiology. In fact, dogs have more abundant tight junctions with a larger pore diameter than humans [32], suggesting a more efficient paracellular absorption [33]. Since C10 also functions as a paracellular permeation enhancer, unlike SNAC, this could help explain the lack of translatability between the two species. This is further supported by a lack of difference in exposure between the two tested meglumine levels in clinic, suggesting that the difference in gastric pH between species might not be the decisive factor contributing to the lack of clinical translation.

However, preclinical data did indeed suggest that C10-based formulations were sensitive to the pH in the gastric milieu. Thus, an alternative hypothesis for the lack of translation could be that the selected amounts of meglumine were insufficient to buffer pH up to the desired level. It is plausible that the amount of meglumine was sufficient in the stomach of acid pre-treated dogs, but still not enough in humans.

On the other hand, it is also noted that a sweet spot may exist regarding the meglumine amount. Excessive meglumine seemed to impair the exposure, as suggested by the reduction of the Cp/D at 0.5 h for the GLP-1 analogue when 85 mg of meglumine was employed instead of 60 mg (Fig. 5A). This has posed significant challenges in precisely determining the necessary amount of meglumine in C10 tablets for achieving optimal peptide exposure.

There could be several reasons why a high amount of meglumine negatively impacts the performance of C10 tablets. First, as pH values increase, the aqueous solubilization of C10 becomes more favourable, which may lead to reduced partitioning of C10 into the cellular membrane. This is due to the change in equilibrium, causing C10 in the membranes (and in vesicular form) to shift towards the aqueous phase. While the transition of colloidal structure from mixed capric acid/caprate vesicles to predominantly caprate micelles at higher pH results in increased availability of monomeric C10 due to the higher critical aggregation concentration (CAC) of the micelles, it is not clear that such colloidal structure is the primary factor driving the permeation-enhancing effect of C10. Berg et al. [34] showed that regardless of the C10 concentration (50, 100 and 300 mM), the ionized C10 in its micellar form (pH 8.5) did not improve the absorption of the model macromolecule FITC-dextran 4000 compared to its vesicle form (pH 6.5) in the rat intestine. More surprisingly, they found that at low C10 concentration (50 mM), the vesicles performed better than the micelles, possibly due to the quick absorption of micellar C10. They concluded that the main drivers of the permeation enhancer effect were most likely the local concentration and the total dose of the enhancer, rather than its colloidal structure. Therefore, having more ionized and micellar C10 at high pH may not necessarily be a merit.

Additionally, a high concentration of meglumine may affect the viscosity of the surrounding environment, potentially hindering API diffusion. However, NMR measurements of equimolar mixtures of meglumine and C10 (200 mg/mL) at 1% to 20% across pH 8, 9, and 10 only showed changes in the chemical shift of meglumine related to pH, with no alterations in line shape or intensity, indicating consistent phase behaviours. Furthermore, viscosity measurements exhibited only a minor increase at 20% meglumine/C10 (Figure S3). Alongside published data on undecanoic acid [35], these results suggest that it is unlikely that viscosity changes significantly impact the performance of the meglumine/C10 formulation.

Finally, higher levels of meglumine may stimulate increased gastric secretions due to the established relationship between stomach pH, gastrin and gastric secretions, which has been studied for nearly 50 years [36–38]. Gastrin release, involving both neuronal and hormonal pathways, promotes HCl secretion in the stomach for food digestion and luminal pH regulation [39]. Although the exact mechanism is not fully understood, a higher gastric pH is known to enhance gastrin release and stimulate acid secretion [40, 41]. Hence, an excessive meglumine level may lead to a high local gastric pH, activating gastric luminal pH sensors and boosting gastric secretions. For instance, in dogs, intramuscular injection of pentagastrine increased gastric volume excretion rates by an average of 13-fold, peaking at 15 min [42]. While the acid output rate is lower in humans than in dogs [43], the efflux from gastric secretions may impede the API absorption [44]. Furthermore, as demonstrated by Chaw et al., elevated gastric pH can induce faster gastric emptying [45], further compromising API absorption which is expected to occur in the stomach, as discussed earlier.

Determining the optimal level of meglumine and assessing the potential benefit of adding a pH-modifier is therefore not straightforward, and further studies should be conducted to elucidate why translation of C10-based formulations from preclinic to clinic can be challenging. As no C10 control formulation was tested in the clinical trial, which only aimed at benchmarking the C10/meglumine formulations against SNAC-based tablets, a definite conclusion regarding the impact of the pH modifier on exposure for C10-based formulations is not possible. Concurrently, whether the increased acidity of the human stomach hampers translation of C10-based immediate release formulations from dogs to humans cannot be conclusively made.

## Conclusion

We developed an immediate-release C10-based formulation incorporating meglumine as a pH modifier, and evaluated it in both preclinical and clinical settings. To enhance the translatability between dogs and humans, we utilized an acid-pretreated dog model and assessed SNAC- and C10-based formulations that had previously undergone clinical trials. The amounts of meglumine and sorbitol were adjusted to ensure optimal pharmaco-technical tablet performance as well as in vivo efficacy. The formulation development involved several model peptides, and the advantages of having the pH modifier were evident across all cases. The lead formulation demonstrated a significant improvement of plasma exposure preclinically compared to the benchmark SNAC formulation. However, the same effect was not reproduced in humans, where the newly developed C10 formulations displayed similar bioavailability to the benchmark SNAC formulation, indicating that the differences in gastric pH between dogs and humans cannot solely account for the lack of translation, and there are other factors influencing the translatability. Further investigations are necessary to identify the underlying causes of this discrepancy, and to establish an animal model that enhances the translatability of C10-containing formulations to humans.

## Materials and methods

### Tablet preparation

SNAC, C10 and APIs used in this study were from Novo Nordisk. Sorbitol and Meglumine EMPROVE^®^ were purchased from Merck (Darmstadt, Germany) and magnesium stearate from Sigma-Aldrich (Saint-Louis, USA).

The PEs were granulated prior to tableting. PE and other excipients were blended with the API by manual geometric mixing, followed by blending in a turbula mixer. Magnesium stearate was added in a secondary blending step prior to compression, also by manual geometric mixing followed by blending in a turbula mixer.

Tablets were produced on STYL’One Evo (Korsch AG) mounted with a single set of punches, and punch size was chosen according to the total tablet weight to well accommodate the powder blends. The press speed was set to 10%. The fill volume was adjusted to obtain tablets having target weights based on composition. Compression forces ranged from 3 to 25 kN to ensure the same apparent density.

### Screening of pH modifiers

Sodium caprate reference material was produced by Novo Nordisk. Analytical grade HCl was purchased from Merck (Darmstadt, Germany). Analytical grade KCl was purchased from Applichem (Darmstadt, Germany). pH modifiers were all purchased from Sigma-Aldrich (Saint-Louis, USA).

A stock solution of KCl 0.1 M/ HCl 0.1 M was prepared and aliquoted in tubes. 20 mL was added per tube, each tube containing 10 mg/mL of C10 granules and gently stirred at 37 °C. The pH modifier was added by small portion until a pH of 9 or above was reached. The amount of pH modifier added for each measurement was preliminary weighted on analytical scale XP 205 (Mettler Toledo). The pH was recorded using a pH meter PHM 200 (Mettler Toledo).

### Screening of the meglumine amount in acidic medium containing C10

Sodium caprate reference material was produced by Novo Nordisk. Analytical grade HCl and Meglumine EMPROVE^®^ were purchased from Merck (Darmstadt, Germany). Analytical grade KCl was purchased from Applichem (Darmstadt, Germany).

A stock solution of KCl 0.1 M/ HCl 0.025 M, pH = 1.6 was prepared. 10mL of this solution was added to 10 mL of MQ water in tubes containing either 50, 100, 200, or 300 mg of C10 granules. Each solution was gently stirred at 37 °C. Meglumine was added in amounts beyond the calculated neutralization point. pH was recorded using a pH meter PHM 200 (Mettler Toledo). Turbidity/clarity was visually assessed for records.

### Viscosity measurements

Equimolar meglumine, caprate solutions were prepared by mixing the compounds in 95% D_2_O/5% H_2_O v/v to give a w/w ratio of the 1:1 complex of meglumine and caprate to water of 1, 5, 10 or 20%, the pH of the solutions was adjusted with HCl or NaOH in H_2_O. The solutions were then transferred to 5 mm Bruker SampleJet NMR tubes and analyzed with a simple ^1^H pulse and acquire experiment (bruker pulse program: zg30 with 16 scans, 2 dummy scans and a 8 kHz sweep width with 64 K data points) on a Bruker 400 MHz NMR system equipped with a Avance IIIHD NanoBay console, a H&F BBO cryoprobe and a cooled SampleJet sample changer. All experiments were conducted at 300 K. After completion of the NMR studies the NMR samples were then used to determine the viscosity of the solutions using Viscoman^™^ (Gilson SAS, France). The principle behind the method is the use of a pipette with a well-defined pipette-tip presumably with a force and pressure measuring device. The crosshead speed and the measured force are used to calculate the shear rate and the shear stress respectively. Viscosity can be expressed in relation to shear rate as:

1 $$\:Viscosity\left(\eta\:\right)=\frac{\tau\:w}{{\upgamma\:}}=\frac{{\Delta\:}{{\rm\:P}}\mathrm{D}/4\mathrm{L}}{32\mathrm{Q}/{\uppi\:}\mathrm{D}3}$$

Where γ is the apparent shear rate, τ_w_ is the shear stress, P is the pressure resulting from driving the plunger, Q is the volumetric flow rate of the fluid passing through the capillary needle, D and L are the internal diameter and length of the capillary. The viscosity (η) is calculated from the obtained shear rate (γ) and shear stress (τ_w_) values by linear regression. The usable range is up to 20,000 mPa.s. The samples were equilibrated 1 h at 37 °C before measuring.

### Small volume disintegration set-up

The disintegration test was performed by a vision-based technique capable of accessing semi quantitatively the amount of tablet that was disintegrated at various time points. In brief, two webcams (BRIO 4 K, Logitech, Lausanne, Switzerland) were fitted into an oven adjusted to 40℃, which corresponded to 37℃ in the disintegration medium. Two webcams were positioned at the side and bottom to record the disintegrating tablet. Two 3700 K LED light strips were mounted in the oven behind a light diffuser illuminating the disintegrating tablets evenly. The webcams were interfaced with an in-house written Python script for frame acquisition. The tablets were placed static in a cuvette of dimension 1.5 × 3.0 × 2.0 cm (depth × width × height). The experiment was started by transferring 5 mL of desired medium, preheated to 37 ℃, into the cuvette followed by acquiring a video from both webcams in a raw [46]. Data analysis was performed using in-house written Matlab scripts (ver. 9.4, Mathworks, MA) together with the Image Processing Toolbox (ver. 11.0, Mathworks, MA).

### Mini-USP2 dissolution

Sodium caprate reference material was produced by Novo Nordisk. Analytical grade KH2PO4, NaOH pellets, Titrisol ampoules 1 N NaOH and HCl, HPLC grade acetonitrile were all purchased from Merck (Darmstadt, Germany). Analytical grade KCl was purchased from Applichem (Darmstadt, Germany). HPLC grade trifluoroacetic acid (TFA) was purchased from Fisher Scientific (Loughborough, UK). Deionized water (18 MΩ) was prepared in the laboratory with a Millipore-Q system (Darmstadt, Germany) for all aqueous solutions. When required, media preparations were pH controlled using Meterlab PHM220 pH meter by Radiometer Analytical (Lyon, France). Two-point calibrations were conducted before media measurement by using reference solutions pH 1.679, 4.005 and 7.000 from Hach (Düsseldorf, Germany).

Small volume dissolution tests of sodium caprate tablets were conducted in sink conditions on a Teledyne Hanson Vision G2 Elite 8 dissolution tester (Chatsworth, CA, US) using a scaled-down USP Apparatus 2, mini paddle and 150 mL vessels (for dimensions see Figure S4). A modified dissolution methodology was used to allow for smaller volumes [47]. Paddle speed was set to 25 rpm, bath temperature was set at 37 °C, and 20.0 mL media volume was used. Dissolution media tested was Medium A (degassed with He, prior tests), two tablet replicates were analyzed per formulation. Medium A was prepared by diluting a solution of 0.025 M HCl, 0.1 M KCl (aq) with H_2_O (aq) in 1:1 (v/v), the final pH of Medium A was pH 1.90 (ionic strength: 0.0625 M). Manual sampling and media addition for volume compensation (Medium A) of 1000 µL were conducted at 5, 10, 15, 20, 30, and 40 min (250 rpm paddle speed between 30 and 40 min). Each sample was filtered through a fresh Millex-GV 13 mm, 0.22 μm PVDF Membrane hydrophilic filter purchased from Merck Millipore (Cork, Ireland), filter prime volume 0.20 mL was used. Each filtered solution sample were then diluted 1:9 with Medium B. Medium B consisted of a mixture of 50 mM KH_2_PO4, 380 mM KCl (aq), pH was adjusted to 7.4 (± 0.05) and the aqueous buffer (ionic strength 0.5 M) was mixed with acetonitrile 20% (v/v) to achieve the final dilution medium (Medium B) composition. The samples were analyzed with High-Performance Liquid Chromatography (HPLC). Dissolution samples were analyzed for sodium caprate recovery using a Waters Alliance HPLC 2695 system (Milford, MA, US) equipped with a quaternary pump, autosampler, temperature-controlled column compartment, and UV detector. The column used was a Waters Sentry Guard Symmetry Shield C8, 100Å, 5 μm, 20 × 3.9 mm. Eluents were A: 0.1% (v/v) trifluoroacetic acid (TFA) in H_2_O and B: 0.1% (v/v) TFA in acetonitrile 80% (v/v) in water. Flow rate was 2.0 mL / min, injection volume 10 µL, run time 4.0 min per injection. Column temperature was 50 °C and UV detection at wavelength 210 nm, autosampler temperature was set to 5 °C throughout the experiment. Linear gradient program used: 25 B% isocratic at 0–1.0 min, 25–80 B% at 1.0–2.5 min, 80 B% isocratic at 2.5–3.0 min, 80–25 B% at 3.0–3.1 min, 25 B% re-equilibration at 3.1–4.0 min. Sodium caprate elutes at retention time 2.80 min. Sample recovery was quantified by using one-point external standard with linear fit through origin as the calibration curve of sodium caprate. The fraction analyte collected at each sampling timepoint was corrected for in the fraction released calculation. Waters Empower software was used for chromatogram processing.

### CAC and CMC determinations

Stock solutions of sodium caprate were made in the individual buffers. 10–100 mM phosphate buffer was used for most sample series, while 50 mM sodium borate was used for pH 9 [31]. In the case of meglumine a 20 mM Tris buffer pH 8.5 was used. Samples were prepared with and without 20 mg/ml meglumine.

All samples were pH adjusted and filtered through 0.22 μm (Millipore 6830-0021). Dynamic light scattering (DLS) data was acquired at 25 °C by adding 20–25 µL of each sample in triplicates to a 384 well plate Corning^®^ 3540 (Corning, NY, USA). The plate was centrifuged for 5 min at 1200 rpm before analysis to remove any air bubbles from the wells. Each well was measured 20–40 times with 5 s acquisition time. A DynaPro™ Plate Reader and Dynamics™ software version 7 (Wyatt Technology Corp., Santa Barbara, CA, USA) was used to collect and analyze the data.

### Preclinical evaluation in beagle dogs

#### Oral administration to beagle dogs

All procedures involving animals were approved by and performed in accordance with the Committee on Animal Care at the Novo Nordisk Environment and Bioethics Committee. Pharmacokinetic (PK) studies in Beagle dogs were conducted to determine the exposure of the therapeutic peptide after peroral administration of different dosage forms. Male Beagle dogs were used, 1 to 7 years of age and weighing approximately 10–16 kg at the start of the studies. The dogs were group housed in pens (12 h light: 12 h dark) and fed individually and restrictedly once. Exercise and group social were permitted daily, whenever possible. The dogs were used for repeated pharmacokinetic studies with a suitable wash-out period between successive dosing’s. An appropriate acclimatisation period was given prior to initiation of the first pharmacokinetic study. All handling, dosing and blood sampling of the animals were performed by trained and skilled staff. Before the studies the dogs were fasted overnight and from 0 to 4 h after dosing. Besides, the dogs were restricted to water 1 h before dosing until 4 h after dosing, but otherwise had ad libitum access to water during the whole period. For the acid pre-treated dogs, oral gavage with the acidic solution (HCl-KCl, pH 1.6) was performed 3 min before oral dosing of the tablet. The tablets containing the therapeutic peptide were administered by placing in the back of the mouth of the dog to prevent chewing. The mouth was then closed, and 10 mL of tap water was given by a syringe to facilitate swallowing of the tablet.

#### Blood sample analysis

Blood was sampled at predefined time points for up till 10 h post dosing to adequately cover the full plasma concentration-time absorption profile of the APIs. Blood samples were taken as appropriate, for example from a venflon in the cephalic vein in the front leg for the first 2 h and then with syringe from the jugular vein for the rest of the time points (the first few drops are allowed to drain from the venflon to avoid heparin saline from the venflon in the sample). For each blood sampling time point approximately 0.8 mL of whole blood was collected in a 1.3 mL EDTA coated tube, and the tube was gently turned to allowing mixing of the sample with the EDTA. Blood samples (for example 0.8 mL) were then centrifuged at 4 °C and 1500 × *g* for 4 min. Plasma was pipetted into Micronic tubes on dry ice and kept at -20 °C until analysis.

The plasma samples from Beagle dogs were analysed for peptide therapeutic using a Luminescence Oxygen Channeling Immunoassay (LOCI). For peptide 1 analysis, a matched antibody pair (2F6 and 3F15) is used in the assay, one biotinylated (3F15) and bound to streptavidin-coated Alpha donor beads, and the other conjugated to AlphaLISA acceptor beads (2F6). The binding of the two antibodies to analyte brings donor and acceptor beads into proximity, resulting in the excitation of donor beads at 680 nm, triggering chemical reactions in the acceptor beads, and subsequently the emission at 615 nm. This emission signal is measured in the EnVision plate reader for API concentration analysis. The amount of light was proportional to the concentration of active peptide ingredient and the lower limit of quantification (LLOQ) in plasma was 500 pM. For analysing PCSK9 inhibitor, the matched antibody pair is 3F15 and 7F1. LLOQ for PCSK) inhibitor was also 500 pM.

Plasma concentrations of GLP-1 analogue were assayed by plasma protein precipitation and analysed by liquid chromatography-mass spectrometry (LC-MS). Calibrators were prepared by spiking blank plasma with analytes to reach the final concentrations in the range from 2 to 1000 nM. Calibrators, plasma blanks or study samples were prepared for LC-MS by adding 1 volume of 8 M Guanidine-HCl and incubated at 37 °C for 30 min. Two and a half volumes of ice-cold methanol containing internal standard were added and mixed on the robot shaker, followed by centrifugation at 4000 rpm at 4 °C for 1 h. The supernatant was diluted with 2 volumes of Milli-Q water containing 1% formic acid before injection on the LC-MS system. The system used was a Transcend II Interface Module SRD3200 system from Thermo Scientific (Waltham, MA, USA) coupled to Orbitrap QExactive Plus mass spectrometer from Thermo Scientific. The LC was equipped with a Cyclone column (CH-953288, Thermo Scientific) as the first dimensional trapping column and Aeris 3.6 μm PEPTIDE XB-C18 as the analytical column (2.1 × 50 mm from Phenomenex). The mobile phase composition of the loading pump is as below: mobile phase A consists of 95% milli-Q water, 2.5% acetonitrile, 2.5% methanol and 1% formic acid; mobile phase B consists of 47.5% acetonitrile, 47.5% methanol, 5% milli-Q water, and 1% formic acid. The analyte of interest was loaded from the Turbo flow column at 15% B to the second dimensional analytical column. The Orbitrap QExactive Plus were operating in positive ionization mode with the parallel reaction monitoring (PRM) scan mode. Linear calibration curves (weighting 1/x^2^) were used for calculating the concentration in the plasma samples. Quality control samples for analytes were included. The deviation between nominal and calculated concentration in the calibrators and quality control samples were below 15% and was below 20% for the LLOQ sample. LLOQ for GLP-1 analogue was 2000 pM. Statistical analysis of the animal data was performed by one-way ANOVO using GraphPad Prism version 9.0.1 (GraphPad Software Inc., La Jolla, California, USA).

### Clinical study

#### Clinical study design

The clinical study was a randomised, open-label, three arm partial parallel-group, single centre (Altasciences Company, Los Angeles, California, USA) study. The study was conducted in accordance with the Declaration of Helsinki and Good Clinical Practice guidelines, and all participants provided informed consent before any trial-related activities. The protocol and informed consent forms were reviewed and approved by appropriate health authorities as well as institutional review boards according to local regulation. The study was registered at ClinicalTrials.gov (identifier: NCT05333107).

In the study, 150 healthy male participants aged 18 to 64 years with a BMI of 20 to 31.9 kg/m^2^ were subjected to once-daily oral administration of 40 mg PCSK9 inhibitor for 15 consecutive days. Participants were randomised to receive one of the three formulations (formulation A, B and C) selected in the preclinical development and the primary endpoint was a 24-h pharmacokinetic profile (AUC_0-24 h_) of PCSK9 inhibitor at day 10 of dosing. All dosing occasions were supervised by qualified site staff and dosing conditions closely monitored. The specific dosing conditions included that participants fasted overnight for a minimum of 6 h, where no fluids were allowed for the last 2 h, before the tablet was administered with 120 mL of water followed by a post-dose fasting period of 30 min. Blood samples for the 24 h pharmacokinetic profile of PCSK9 inhibitor were collected continuously throughout the time window and PCSK9 inhibitor was measured by a validated assay using plasma protein precipitation with LC-MS/MS detection.

#### Blood sample and LC-MS analysis

For each blood sampling time point, blood was collected in a 3.0 mL K3 EDTA tube, and the tube was gently turned to allowing mixing of the sample with the EDTA. Blood samples were then centrifuged at 2–8 °C at 1500 x g for 10 min. Plasma was pipetted into 3.6 mL Nunc Cryotubes and kept at -20 °C until analysis.

Plasma concentrations of PCSK9 inhibitor were assayed by a validated method using plasma protein precipitation and analysed by liquid chromatography-mass spectrometry (LC-MS). Calibrators were prepared by spiking blank plasma with analytes to reach the final concentrations in the range from 5 to 500 nM. All calibrators, plasma blanks or study samples were vortexed and mixed 2:1 with 100 nmol/L stable isotope labelled internal standard except blanks. Samples were mixed by shaking for 5 min 750 rpm at room temperature before mixing 1:4 with acetonitrile /methanol (50:50 v/v). Following this, samples were mixed by shaking for 10 min at 1000 rpm at room temperature before centrifugation at 4000 rfc for 15 min, 5 °C. The supernatant was diluted 2:1 with Milli-Q water before injection on the LC-MS system.

The system used was a Triple Quad 6500 system with electrospray ionization (ESI). The LC was equipped with an XSelect CSH C18 CP analytical column (2.1 × 50 mm) from Waters. The mobile phase composition of the loading pump is as below: mobile phase A consists of 95% milli-Q water, 5% acetonitrile, and 1% formic acid; mobile phase B consists of 95% acetonitrile, 5% milli-Q water, and 1% formic acid; mobile phase C consists of MilliQ Water added 1% formic acid.

The Triple Quad 6500 system was operating in positive ionization mode with the multiple reaction monitoring (MRM) scan mode.

Baseline integration was performed using Analyst software v.1.7.1 (or higher) and linear calibration curves (weighting 1/x2) were used for calculating the concentration in the plasma samples using Watson LIMS v.7.6.1 (or higher). Quality control samples for analytes were included. The deviation between nominal and calculated concentration in the calibrators and quality control samples were below 15% and was below 20% for the LLOQ sample. LLOQ for PCSK9 inhibitor was 5 nM.

#### Statistical analysis

Statistical analysis of the animal data was performed using GraphPad Prism version 9.0.1 (GraphPad Software Inc., La Jolla, California, USA).

Statistical analysis of the clinical data was performed using SAS version 9.4 (SAS Institute, Cary, North Carolina, USA). The sample size of the clinical study was selected to ensure at least 80% power if the true difference between formulations is 50%. The primary endpoint (AUC_0-24 h_) was assumed to be log-normal distributed with a common standard deviation of 0.675 on the log-scale. Based on these assumptions, at least 45 completed participants were needed for each of the tablet formulations. The primary endpoint (AUC_0-24 h_) was calculated using the linear trapezoidal method based on observed plasma concentrations of PCSK9 inhibitor and the actual measurement times. The AUC_0-24 h_ was log-transformed and analysed in an ANOVA model with formulation as fixed effect. No adjustment for multiplicity was performed.

## Electronic supplementary material

Below is the link to the electronic supplementary material.

Supplementary Material 1

## Data Availability

All data associated with this study are present in the paper or in the Supplementary Materials. Raw data and reagents may be made available upon reasonable request from Novo Nordisk A/S under a materials transfer agreement.

## Data availability

Raw data are available upon reasonable request.
