# Supplementary material for: An effort to enhance the clinical translatability of caprate-based tablet formulations in gastric peptide delivery
Source: Drug Deliv Transl Res. 2025 Sep 23;16(7):2302–15. doi: 10.1007/s13346-025-01978-7 (PMC13294250; doi:10.1007/s13346-025-01978-7)
Supplement: Supplementary file 1 — Supplementary Material 1 [file 13346_2025_1978_MOESM1_ESM.docx]

**Supporting Information**

**Table S1.** Calculated final pH in dog stomach after oral gavage of 10mL 0.025M HCl solution, according to dogs’ body weight and initial stomach pH

| Dog body weight (kg) | Initial pH in dog stomach | Final pH once mixed with 10ml of 0.025M HCl* |
| --- | --- | --- |
| 10 | 3.00 | 1.6 to 1.8 |
| 16 | 8.00 | 1.6 to 1.9 |

* Calculation made assuming 0.1 to 0.6 mL/kg of gastric juice in the stomach

**Table S2.** PK results of tablets containing 5 mg of GLP-1 analogue and 100 mg of C10 with variable amounts of meglumine and sorbitol orally dosed in beagle dogs (acid pre-treated dogs). Data expressed as mean ± SEM, N = 16.

| Formulation | Meglumine (mg/tablet) | Sorbitol (mg/tablet) | Cp/D_0.5h (kg/L) |
| --- | --- | --- | --- |
| Comparator: 300 mg SNAC (no C10) | - | - | 0.26 ± 0.05 |
| C10 control | - | 8* | 0.13 ± 0.02 |
| C10/Meglumine control | 35 | 8* | 0.26 ± 0.06 |
| C10/Sorbitol control | - | 43 | 0.31 ± 0.04 |
| C10/Sorbitol/Meglumine  (lead formulation) | 60 | 40 | 0.44 ± 0.07 |
| C10/Sorbitol/Meglumine  (higher Meglumine level) | 85 | 40 | 0.38 ± 0.04 |

* A minimum amount of 8 mg of sorbitol/tablet was found to be necessary for
manufacturability reasons. Cp: plasma concentration; D: dose; SEM: standard error of mean.

A

B

| Peptide | Molecular weight (Da) | Isoelectric  Point | ALogP |
| --- | --- | --- | --- |
| GLP-1 analogue | 4888 | 4.0 | -11.83 |
| PCSK9 inhibitor | 5930 | 4.1 | -16.27 |

**Figure S1.** (A) Chemical structure of a GLP-1 analogue and (B) physicochemical properties of the GLP-1 analogue and a PCSK9 inhibitor evaluated in this study.

**Figure S2.** Screening of different pH modifiers in a 20 mL acidic media (0.1M HCl/0.1M KCl), containing 10 mg/mL of C10, under gentle stirring at 37°C

**Figure S3.** Viscosity measurement of solutions of equimolar mixtures of meglumine and C10 at pH 8, 9, and 10.

**Figure S4.** Dissolution dimensions of scaled-down USP 2 vessel and paddle.
